# Asthma Risk Associated with Indoor Mold Contamination in Hispanic Communities in Eastern Coachella Valley, California

**DOI:** 10.1155/2018/9350370

**Published:** 2018-10-15

**Authors:** Ryan Sinclair, Charity Russell, Genevieve Kray, Stephen Vesper

**Affiliations:** ^1^Environmental Microbiology Research Laboratory, Loma Linda University School of Public Health, B2E Evans Hall: 24785 Stewart Street, Loma Linda, CA 92354, USA; ^2^National Exposure Research Laboratory, United States Environmental Protection Agency, 26 West M. L. King Drive, Cincinnati, OH 45268, USA

## Abstract

Indoor mold contamination has been associated in many studies with an increased risk of asthma and respiratory illness. This study investigated indoor mold contamination and the prevalence of asthma/respiratory illness in two low-income, Hispanic communities, Mecca and Coachella City, in the Eastern Coachella Valley (ECV) of California. The study consisted of a questionnaire to assess asthma/respiratory illness and the quantification of mold contamination in house dust samples using the Environmental Relative Moldiness Index (ERMI) scale. About 11% of the adults and 17% of the children in both Mecca and Coachella City met our definitions of asthma/respiratory illness. The average ERMI values in Mecca and Coachella City housing (10.3 and 6.0, respectively) are in the top 25% of ERMI values for the United States (US) homes. Overall, the homes surveyed in these ECV communities had an average prevalence of occupant asthma of 12.8% and an average ERMI value of 9.0. The prevalence of asthma/respiratory illness in the Hispanic communities of Mecca and Coachella City and the mold contamination in their homes appear to be greater than the averages for the rest of the US. The higher levels of mold contamination in their homes appear to be associated with a greater risk of asthma/respiratory illness for these low-income, Hispanic communities.

## 1. Introduction

**Figure 1:**
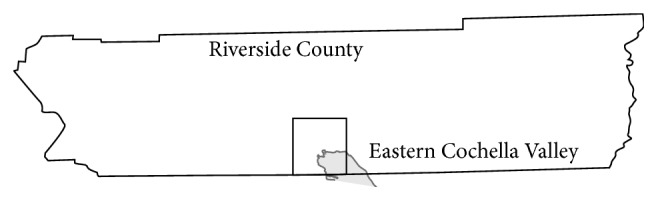
Eastern Coachella Valley shown (square) on map of Riverside County California.

The prevalence of asthma nearly doubled in the United States (US) between 1980 and 1995, but between 2001 and 2010, the increase was more gradual, and for most demographic groups, the prevalence of asthma seems to have leveled off or declined slightly [1]. However, there is one group in which the prevalence of asthma continues to rise significantly: the poor, defined as family income below the Federal Poverty Level [1]. Most families that live in the Eastern Coachella Valley (ECV) in Riverside County, California (Figure 1) would be defined as poor.

**Figure 2:**
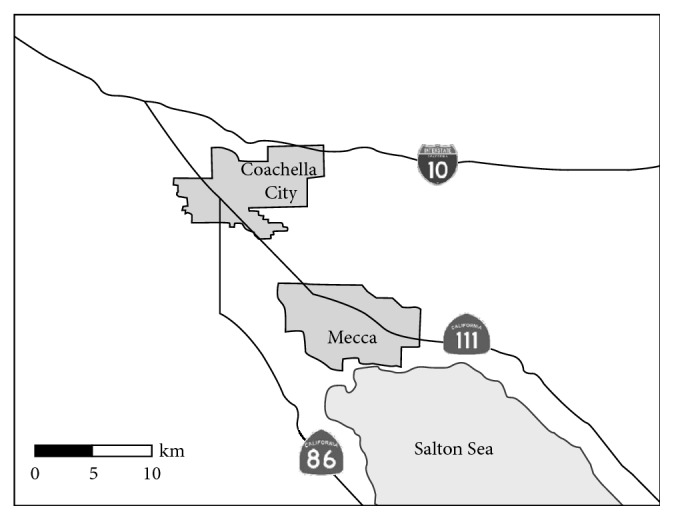
Map of the communities of Coachella City and Mecca in the Eastern Coachella Valley.

The California Health Institute (CHI) performed a phone survey of the health conditions throughout the State in 2014 [2]. Based on this phone survey, the prevalence of asthma in the ECV was assessed at about 12.7% (95% confidence intervals, 7.6–17.8%) [2]. In 2015, the California Institute for Rural Studies (CIRS) and Loma Linda University (LLU), in partnership with the California Endowment, launched a comprehensive regional health survey of the ECV communities, including Mecca and Coachella City (Figure 2).

Asthma prevalence is often high in poor, urban communities [3]. However, there are large populations of poor living in rural areas. These rural poor are frequently Mexican Hispanic, often agricultural workers and their families [4]. Whereas in Mexico, the prevalence of asthma in Hispanic children is low [5]; if they move to the US as children, their asthma risk increased significantly [6]. Jerschow et al. found that among foreign-born Mexicans, rates of asthma were greater after relocation versus before relocation (adjusted hazard ratio 2.90 for after versus before relocation) [7]. In a study of asthma in Hispanic children living in the Arizona-Mexico border region, the adjusted odds ratio (OR) for asthma was significantly higher (OR = 4.89) in the US Hispanic population compared with the children in Mexico [8]. Although the authors suggested that there might be some factor in Mexico that protects children from asthma, it could also be hypothesized that something present in US homes promotes asthma.

It has been known for many years that exposure to damp/moldy buildings increases the risk of asthma and respiratory illness [9, 10]. However, accurate quantification of mold exposures has been limited by the traditional methods used to quantify mold exposures, e.g., short air samples that are counted or cultured [11]. Therefore, the US EPA, in conjunction with the US Department of Housing and Urban Development, developed the Environmental Relative Moldiness Index (ERMI) scale [12]. A panel of 36 indicator-molds is quantified using DNA-based assays [12]. The ERMI scale ranges from about −10 to about 30, i.e., lowest to highest mold contamination. The ERMI methodology has been used in six previous epidemiological studies of asthma, and higher ERMI values were consistently associated with asthma development and/or exacerbation [13, 14].

Our first goal was to try to obtain an accurate estimate of asthma/respiratory illness for both adults and children based on the California Institute for Rural Studies questionnaire, using multiple questions to make the assessment. Our second goal was to examine the different types of housing in Mecca and Coachella City to determine the level of mold contamination in the various types of housing in these communities, as defined by their ERMI values and the relationship between the ERMI values and the prevalence of asthma/respiratory illness.

## 2. Materials and Methods

This study was conducted in accordance with the ethical principles set forth in the Declaration of Helsinki of the World Medical Association, and the protocol was approved by the Loma Linda University Institutional Review Board (IRB #5140048). The Loma Linda University IRB is registered with the US Office for Human Subject Research Protection (IRB#0000383).

### 2.1. Study Design

A major population of the ECV is migrant, farmworker families that may not be responsive to typical phone or Internet-based health assessments due to issues ranging from (1) the remote location of the household, (2) the informal nature of the house that they reside in, and (3) not being listed on any publicly available phone or address list. Therefore, this study attempted to compensate for those factors by using extensive on-the-ground observation prior to creating a sampling frame for the in-person surveys. Before sampling, the study team reviewed satellite images to define groups of dwellings and the geographic limits of these strata. A “ground-truthing” walk occurred after this, where the field research team walked the limits of the strata to validate the locations of the communities and households that would be within the sampling frame. The result was a new community map that allowed a systematic, random sample to be obtained.

The survey administrators were then able to give teams of Loma Linda University students and local community health “promotoras” lists of preselected addresses that were chosen using a randomized cluster sampling method. No house substitutions were allowed, and surveyors validated the house location through visits from the survey supervisor, and a GIS device was used during the time of the survey. The result was randomly selected households in the communities of Mecca (*n*=342) and Coachella City (*n*=353) (Figure 2). The survey and sampling methods were approved by the Loma Linda University Institutional Review Board.

The asthma section of the California Institute for Rural Studies questionnaire was adapted from previous (validated) Spanish language farmworker assessments and validated again using external consultants and internal stakeholders [15]. The questions related to asthma diagnosis, treatment, and/or symptoms were used to establish the prevalence of asthma/respiratory illness in the communities. Adults who answered “yes” to any of the five following questions were categorized as positive for asthma/respiratory illness. (1) Have you ever been diagnosed with asthma? (2) Are you currently being treated for asthma? (3) Have you had an asthma attack severe enough to limit activity? (4) Are you currently taking asthma medication? (5) Do you have daily, weekly, or severe cough?

If the parent or guardian answered “yes” to any of the four following questions, the child was categorized as positive for asthma/respiratory illness. (1) Has your child ever been diagnosed with asthma? (2) Are you able to obtain asthma medication for your child? (3) For the child with asthma, have you ever had to take the child to the emergency room for their asthma? (4) Does your child have a persistent cough?

### 2.2. Types of Housing

The housing in the communities was divided into four types: apartments, modern homes, trailers, and mixed-use. The classification of housing type for each respondent was made by the survey team. An apartment was defined as a structure with multiple families in individual units. A modern home was defined as a single-family home built after 1990 (as determined from city development records). A trailer was defined as a potentially mobile structure that sits upon two axels and jack stands that are hidden from view using foundation panels. A mixed-use residence was defined as a home with other informal housing units built within the household's single-family parcel boundaries, including rental rooms in outbuildings for seasonal farmworkers.

### 2.3. Dust Sampling and Analysis

A subset of homes was sampled for mold analysis when the occupant gave permission. A dust sample was collected in each home in the subset in Mecca (*n*=50) and Coachella City (*n*=61) by wiping the tops of doorways, bookshelves, and other above floor surfaces using a Swiffer™ sweeper cloth (P&G, Cincinnati, OH) [16]. The survey team member collecting the dust sample wore a disposable glove to avoid contaminating the sample. After collection of the dust sample, the cloth was placed in a zippered plastic bag and labeled with the study number. Samples were kept at room temperature until returned to the lab, where the samples were frozen at −20°C until analyzed.

Each dust sample was sieved (300 *µ*m pore size) and 5 mg of each sieved dust sample was extracted to recover the DNA, which was then purified using the DNA-EZ kit (Generite, Monmouth Junction, NJ). Each of the 36 ERMI molds was quantified by mold-specific quantitative PCR (MSQPCR) assays [17].

The ERMI metric classifies the 36 indicator-mold species into either Group 1, the 26 species indicating water damage, or ten Group 2 species which are commonly found in homes across the US, even without water damage, and come primarily from outdoors [11]. The ERMI calculation takes the results from the concentrations (cell equivalents/mg dust) of each of the 36 molds and mathematically converts these into a single number as shown in the following equation:

(1) $$\begin{array}{c} \text{ERMI}=\overset{26}{\underset{i=1}{\sum }}\log_{10}\left(s_{1i}\right)-\overset{10}{\underset{j=1}{\sum }}\log_{10}\left(s_{2j}\right). \end{array}$$

The concentration of each of the 26 Group 1 molds is converted to a log, and then the “Sum of the Logs of the Group 1” (*s*_1*i*_) molds is determined. Similarly, the concentration of each of the ten Group 2 molds is converted to a log and then the “Sum of the Logs of the Group 2” (*s*_2*j*_) molds is determined. The arithmetic difference, *s*_1*i*_ − *s*_2*j*_, is the ERMI value for the home [12]. Therefore, the higher the ERMI value, the greater the mold contamination in the home.

### 2.4. Statistical Analysis

Before analysis, all data were deidentified, with addresses and names removed from the database. The statistical difference between the average ERMI values in Mecca and Coachella City was evaluated using Student's *t*-test. The statistical analysis of the differences in concentrations of 36 individual mold species in Mecca and Coachella homes was evaluated with the Mann–Whitney rank sum test. These differences were then corrected for multiple comparisons using the Holms–Bonferroni test. All analyses were performed in SAS version 9.3 (SAS Institute, Cary NC) or R version 2.14 (R Foundation for Statistical Computing, Vienna, Austria).

## 3. Results

The respondents' answers to the questions about national identity, gender, age, income, and occupation are summarized in Table 1. Both communities were about 98% Hispanic. About 85% of the families reported yearly income < $20K/year. About 40% of men and 22% of women from both Mecca and Coachella City worked in agriculture.

The percentage of adults and children that met our definition of asthma/respiratory illness in the ECV communities was about 11 and 17.5%, respectively (Table 2). The overall prevalence of asthma/respiratory illness was highest for children living in mixed-use housing in both Mecca (32.3%) and Coachella City (40%) (Table 2). Adults living in trailers in Mecca had the highest percentage with asthma/respiratory illness, but adults living in mixed-use housing in Coachella City had the highest percentage with asthma/respiratory illness (Table 2).

The average ERMI value in Mecca housing (10.3) was significantly greater than the average ERMI value in Coachella City housing (6.0) (Table 3). Trailers and mixed-use housing in Mecca had average ERMI values nearly twice as high as for apartments or modern homes in Mecca (Table 3). In Coachella City, all four types of housing had similar average ERMI values.

The prevalence of asthma/respiratory illness for occupants (both adults and children together) was compared with the combined average ERMI values for Mecca and Coachella City (Table 4). Mixed-use housing had the highest percentage of occupants with asthma/respiratory illness (17.4%) and the highest average ERMI values (13.2), and apartments had the lowest percentage of occupants with asthma/respiratory illness (9.3%) and the lowest average ERMI value (5.9). Overall, the homes surveyed in the ECV communities of Mecca and Coachella City had an average prevalence of occupant asthma of 12.8% and an average ERMI value of 9.0 (Table 4).

The populations of each of the 36 ERMI molds were compared in Mecca vs. Coachella City housing to determine if there were any significant differences (Table 5). The populations of four of the Group 1 molds (*Aspergillus ochraceus*, *A. sydowii*, *A. versicolor*, and *Stachybotrys chartarum*) were in significantly greater numbers in residences in Mecca compared with Coachella City. None of the populations of Group 2 molds were significantly different in Mecca and Coachella City housing.

## 4. Discussion

The Centers for Disease Control and Prevention reported that the prevalence of asthma in the US as a whole was 7.6% for adults and 8.4% for children based on the National Health Interview Survey (NHIS) questions: for adults, “Have you ever been told by a doctor or other health professional that you had asthma?” and “Do you still have asthma?”; and for children, “Has a doctor or other professional ever told you that [sample child] had asthma?” and “Does [sample child] still have asthma?” [18]. It is difficult to make an accurate assessment of asthma's prevalence in the Eastern Coachella Valley, since many residents do not receive medical care. Our estimate of asthma/respiratory illness in each community separately was 11% for adults and 17.5% for children based on the answers to our questionnaire. The overall estimate of asthma/respiratory illness for adults and children in these ECV communities was 12.8% (Table 4). This is consistent with the overall estimate from the CHIS phone survey of 12.7% (95% confidence intervals, 7.6–17.8%) in ECV [2]. Therefore, our multiquestion approach to defining asthma/respiratory illness appears to be reasonable and suggests we have surveyed representative populations in each community.

The average ERMI values for the four types of homes in the Eastern Coachella Valley (*n*=111) ranged from 5.9 to 13.2 with an overall average of 9.0 (Table 4). In the broader context, the homes (*n*=17) randomly selected from Riverside County California during the 2006 HUD American Healthy Homes Survey had an average ERMI value of 1.97 [12]. This suggests that mold contamination, in general, was much greater in the Eastern Coachella Valley housing compared with housing in Riverside County generally.

There are a several likely sources of the water damage in ECV housing that could lead to mold growth, although the specific sources of water problems for each home in this study were not determined. Plumbing leaks can occur in any type of housing, but older homes are more likely to experience plumbing leaks. A second possible source of water problems is “swamp coolers.” Swamp coolers are common in ECV housing. Evaporative cooling by swamp coolers works by bringing humidified air into the home. If swamp coolers are not well maintained, they can lead to excess moisture and mold growth. Another source of water problems is precipitation. Even in a desert region, there are rain events, some even causing flooding. Home maintenance and repair are critical to preventing mold growth from rain.

Governmental and international health organizations have reported a link between water damage and indoor mold contamination and asthma or poor respiratory health [9, 10]. In six previous epidemiological studies, homes with higher ERMI values were associated with occupant asthma [13]. For example, in a prospective epidemiological study, pregnant women were enrolled and the home's environment and the child's health were monitored for seven years, until a physician could make a diagnosis of asthma. Settled dust collected in each infant's home was analyzed for mold contamination based on its ERMI value. Infants living in homes with high ERMI values, i.e., in the top 25% of US homes (ERMI > 5.2) were at more than twice the risk of developing asthma than those in lower ERMI value homes [14]. Three molds were associated with asthma development in this prospective study [19], and one of the three molds was *Aspergillus ochraceus*, which was found to be in significantly higher concentrations in Mecca housing compared with Coachella City. However, these studies do not prove that the mold itself was the cause of asthma. However, the mold contamination, measured as ERMI values, appears to be correlated with conditions in the home related to occupant asthma.

There are many limitations to this study. It was not possible to obtain a definitive diagnosis of asthma based on a physician's evaluation of each adult or child. These are transient populations with little access to medical care. Because these populations are transient, people living in the same home may or may not be genetically related. Therefore, we were not able to consider atopy as an important confounder in the analysis. Another significant limitation to this study was that we relied on the respondent's answers to the questionnaire to assess who had asthma. However, we did use multiple questions to assess asthma diagnosis from different angles. For adults, we asked about whether the adult had ever been diagnosed with asthma, and we also asked about current symptoms and asthma medications. Unfortunately, many of the children have never seen a physician and some may be in the country illegally. This is particularly a problem when “guardians” responded about children living in the home. Therefore, we cannot directly link the home to current asthma.

Other limitations include the fact that other potential exposures inside and outside the home were not quantified. Also, only a relatively small number of each of the housing types was tested in each community. Despite these acknowledged limitations, these results provide evidence that conditions in Mecca and Coachella City homes are conducive to an increased risk of asthma/respiratory illness and emphasize the need to improve the environmental conditions in homes [20].

## 5. Conclusions

The housing in the ECV communities of Coachella City and Mecca had ERMI values that placed them in the top 25% of homes in the US. It also appears that the prevalence of asthma in the communities of Mecca and Coachella City is much higher than that in the US generally.

## Figures and Tables

**Table 1:** Demographic characterization of the communities of Mecca and Coachella City in Eastern Coachella Valley, California.

	Mecca percentage	Coachella City percentage
National identity		
Hispanic	98	98
Others	2	2

Gender		
Male adult	48	46
Female adult	52	54
Age (mean and standard deviation)		
Adult male (years)	41 + 17	41 + 17
Adult female (years)	39 + 14	41 + 16

Family income		
<$10K	46	50
$10–20K	39	37
$20–30K	12	7
>$30K	3	5

Occupation		
Male adult		
Agriculture	41	24
Others	33	45
None listed	26	31
Female adult		
Agriculture	39	20
Others	26	42
None listed	35	37

**Table 2:** Mean percentage of children or adults that were assessed as positive for asthma/respiratory illness (%A/RI) in Mecca and Coachella City and the percentage of type of homes (%TH) sampled in Mecca (total *n*=342) or Coachella City (total *n*=353) that were occupied by a child or adult.

	Mecca				Coachella City			
	Children		Adults		Children		Adults	
	% A/RI (% TH)		% A/RI (% TH)		% A/RI (% TH)		% A/RI (% TH)	
Apartment	18.3	(54)	13.1	(31)	19.0	(21)	8.8	(26)
Modern home	17.9	(12)	5.4	(27)	17.7	(46)	12.7	(47)
Trailer	6.1	(21)	14.5	(22)	13.4	(29)	9.0	(25)
Mixed-use	32.3	(13)	12.1	(20)	40.0	(4)	42.9	(2)
Overall mean %	17.5		11.1		17.5		11.3	

**Table 3:** Average Environmental Relative Moldiness Index (ERMI) values in homes in the four types of housing in Mecca and Coachella City and the weighted-average ERMI values (WAEV) for all housing in each community. (*n* = number homes evaluated in each category).

	Mecca		Coachella City		
	*n*	ERMI	*n*	ERMI	*p* value
Apartment	16	7.1	17	4.9	
Modern home	18	7.8	22	6.1	
Trailer	7	17.2	18	7.0	
Mixed-use	10	15.3	3	5.9	
Total	51		60		
WAEV		10.3		6.0	<0.05

**Table 4:** The Mecca and Coachella City data were combined to determine the correlation between the total percentages of both adults and children assessed with asthma/respiratory illness in the four types of Eastern Coachella Valley housing and the corresponding average Environmental Relative Moldiness Index (ERMI) values in the four types of Eastern Coachella Valley housing.

	Total % illness	Average ERMI
Apartment	9.3	5.9
Modern home	13.0	6.9
Trailer	11.2	9.8
Mixed-use	17.4	13.2
Average	12.8	9.0

**Table 5:** Comparison of the average (AVG) concentration, as cell equivalents (CE) per mg of dust, of the 36 Environmental Relative Moldiness Index mold populations in the residences in Mecca and Coachella, California.

	Mecca AVG CE/mg dust	Coachella AVG CE/mg dust	Wilcoxon *p* value^*∗*^
Group 1 molds			
*Aspergillus flavus*	1221	29	0.55
*Aspergillus fumigatus*	361	36	0.57
*Aspergillus niger*	1668	964	0.005
*Aspergillus ochraceus*	**229**	**12**	**<0.001**
*Aspergillus penicillioides*	44	25	0.18
*Aspergillus restrictus*	3	2	0.42
*Aspergillus sclerotiorum*	0	0	0.38
*Aspergillus sydowii*	**22027**	**20**	**<0.001**
*Aspergillus unguis*	555	10	0.19
*Aspergillus versicolor*	**748**	**8**	**<0.001**
*Aureobasidium pullulans*	126	133	0.72
*Chaetomium globosum*	4	2	0.96
*Cladosporium sphaerospermum*	113	26	0.47
*Eurotium amstelodami*	2347	187	0.001
*Paecilomyces variotii*	74	15	0.002
*Penicillium brevicompactum*	11	9	0.11
*Penicillium corylophilum*	17	165	0.92
*Penicillium crustosum*	154	7	0.13
*Penicillium purpurogenum*	5	6	0.92
*Penicillium spinulosum*	0	0	0.92
*Penicillium variabile*	17	7	0.85
*Scopulariopsis brevicaulis*	279	3	0.11
*Scopulariopsis chartarum*	193	9	0.11
*Stachybotrys chartarum*	**123**	**4**	**<0.001**
*Trichoderma viride*	0	5	0.9
*Wallemia sebi*	90	55	0.02

Group 2 molds			
*Acremonium strictum*	1	1	0.9
*Alternaria alternata*	2576	79	0.009
*Aspergillus ustus*	1719	6	0.24
*Cladosporium cladosporioides* 1	82	98	0.94
*Cladosporium cladosporioides* 2	123	60	0.52
*Cladosporium herbarum*	155	209	0.16
*Epicoccum nigrum*	85	115	0.46
*Mucor group*	48	22	0.12
*Penicillium chrysogenum* 2	6057	25	0.11
*Rhizopus stolonifer*	71	22	0.26

^*∗*^The statistical analysis of the differences in concentrations of 36 individual mold species was evaluated by the Mann–Whitney rank sum test. These differences were then corrected for multiple comparisons using the Holms–Bonferroni test. Only molds bolded were significantly different.

## Data Availability

The data used to support the findings of this study are available from the corresponding author upon request.
